# Hardening and Softening Behavior of Caliber-Rolled Wire

**DOI:** 10.3390/ma15082939

**Published:** 2022-04-18

**Authors:** Joong-Ki Hwang

**Affiliations:** School of Mechatronics Engineering, Korea University of Technology & Education, Cheonan 31253, Korea; jkhwang@koreatech.ac.kr; Tel.: +82-041-560-1642

**Keywords:** Bauschinger effect, caliber rolling, strain hardening, wire drawing

## Abstract

The different behaviors of the mechanical properties of drawn and caliber-rolled wires with applied strain were investigated to determine the appropriate process between wire drawing and caliber rolling with consideration of materials and process conditions. Ferritic, pearlitic, and TWIP steels were drawn and caliber-rolled under the same process conditions. Caliber-rolled wires exhibited a hardening behavior in the early deformation stage and softening behavior in the later deformation stage compared with the drawn wires, regardless of the steel. The hardening behavior of the caliber-rolled wires was explained by the higher strain induced by caliber rolling compared with wire drawing, especially the higher amount of redundant work in caliber-rolled wire. The caliber-rolled wire had approximately 36% higher strain than the drawn wire and approximately 85% higher strain than nominal strain. The softening behavior of the caliber-rolled wire in later deformation stages was related to the Bauschinger effect or low-cycle fatigue effect caused by the roll geometries and loading conditions during caliber rolling. The different intersection points of the tensile strength between drawn and caliber-rolled wires with the steels were attributed to the different strain hardening rates of each steel. Between the options of the caliber rolling and wire drawing processes, the appropriate process should be selected according to the strain hardening rate of the material and the amount of plastic deformation. For instance, when the wires need to deform at high levels, wire drawing is the better process because of the appearance of the Bauschinger effect in caliber-rolled wire.

## 1. Introduction

**Figure 1:**
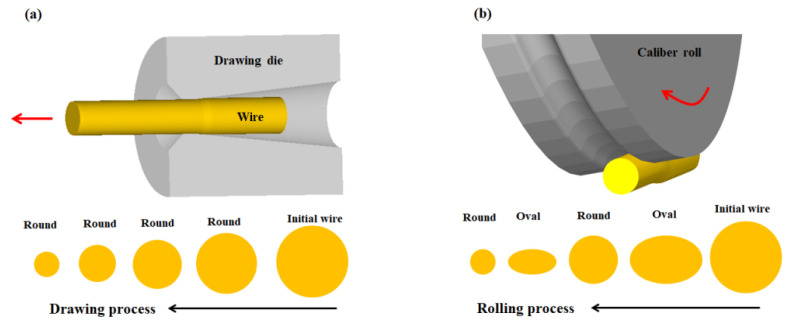
Schematic of (**a**) wire drawing and (**b**) caliber rolling processes used in this study.

Rolling is the most important metal forming process in industrial fields. More than 90% of steel, copper, aluminum, and their alloys undergo a rolling process at least once, owing to the high productivity and low costs of the rolling process. The rolling process is classified into hot rolling and cold rolling, depending on the working temperature. In addition, based on the product shape, rolling can be categorized into plate rolling and shape rolling [1]. In plate rolling, both the cold rolling and hot rolling are widely used in the metal forming industry. In contrast, in industrial fields, hot shape rolling is widely used, but cold shape rolling is not. Recently, Hwang [2] suggested that cold caliber rolling with an oval-round sequence can replace the wire drawing process owing to several advantages of the caliber rolling process over the wire drawing process. For example, the caliber rolling process changes the frictional mechanism at the specimen and tool interface from sliding to rolling, and the rolling process changes the main stress state from tension to compression, as shown in Figure 1, leading to a decrease in the wire breakage rate during the process, and an increase in the reduction ratio per pass compared with wire drawing. Additionally, prior to the rolling process, specimen preparation was relatively simple.

Despite the advantages of the caliber rolling process compared with the wire drawing process, few studies exist on the caliber rolling process at cold and warm rolling temperatures [2,3,4,5,6,7,8,9,10]. Yin et al. [3] revealed that a caliber-rolled ferritic steel exhibits a <110> fiber texture at the recrystallization temperature. Inoue et al. [4] investigated the relationship between the strain and microstructural distributions in plain low-carbon steel during warm caliber rolling. They showed that inhomogeneous strain distribution in the cross-section of the caliber-rolled wire leads to microstructural inhomogeneity. Hwang and Kim [10] reported that the strain inhomogeneity of caliber-rolled wires can be improved using skin pass caliber rolling. Reuter et al. [5] reported that the tensile strength (TS) of a caliber-rolled wire processed at room temperature (RT) was lower than that of a drawn wire by comparing the strength of pearlitic steel with 0.71 (wt.%) carbon content. Krallics et al. [8] fabricated ultrafine-grained titanium by caliber rolling at warm temperature. They demonstrated that caliber-rolled titanium exhibits high strength and good ductility. Chen et al. [9] reported that caliber-rolled TWIP steel wires showed a better combination of strength, ductility, and hydrogen embrittlement resistance than the conventional wire rod steels. Recently, several researchers have focused on caliber rolling to improve the strength-ductility balance, toughness, and fatigue properties of metals, such as medium-carbon steel [11], hypereutectoid steel [12], Zn alloys [13], Mg alloys [14], Ti alloys [15], and composite materials [16,17].

In the previous study of the authors [2], it was revealed that caliber-rolled TWIP steel wire exhibits higher strength than the drawn wire at the same applied strain. Based on these results, it was suggested that caliber rolling can replace the wire drawing process. This study can open a new gate to the application of the cold caliber rolling process in the wire, rod, and bar industries; however, it is somewhat difficult to derive a general conclusion because the results were obtained after testing only one microstructure and the applied strain was limited. Accordingly, the ferritic and pearlitic steels were additionally selected for the general conclusion of wire drawing and caliber rolling behaviors because these two steels have completely different microstructures from the TWIP steel, and they are widely used in conventional wire, rod, and bar products. Furthermore, deformation behaviors of the three steels were compared after conducting wire drawing and caliber rolling tests.

This experiment showed that the behaviors of the mechanical properties in drawn and caliber-rolled wires were different with respect to the microstructures, indicating that the appropriate process is different for each material. Additionally, the caliber-rolled wire showed hardening and softening behaviors with applied strain compared with the drawn wire, which has not been reported in the literature. This result stimulated an additional investigation on this research topic. The author believes that the softening behavior of the caliber-rolled wire was highly related to the Bauschinger effect (BE) and/or low-cycle fatigue effect due to the roll geometry and loading conditions during the caliber rolling process (Figure 1). To the best of our knowledge, the BE in the caliber rolling process has never been reported because shape rolling with an oval-round sequence is most commonly used in the hot rolling process.

Meanwhile, a decrease in the yield stress was well-reported in the pipe forming process such as UOE pipe forming and spiral pipe forming [18,19,20,21,22] and cold tube rolling [23,24]. Kostryzhev et al. [18] reported the BE of a plate during the UOE line pipe forming process with a large diameter. They showed that the yield stress increased owing to the strain hardening of material, and decreased in reverse loading owing to BE. Ren et al. [19] investigated the UOE pipe forming process using a finite element method incorporated with a kinematic hardening model to describe the BE of X80 steel. Zhang et al. [20] predicted the difference in yield stress between UOE pipe and initial plate of a low-carbon micro-alloyed steel. They found that the yield stress difference of pipe and plate is the result of the combined action of the BE and strain hardening during the pipe forming process. In addition, the yield stress was revealed to be dependent on the ratio of pipe wall thickness to pipe outer diameter. For example, the BE is dominant when the pipe thickness is small and pipe diameter is large. Sohn et al. [21] investigated the influence of the microstructure and forming strain of API X70 and X80 line pipe steels on yield stress during spiral pipe forming. They reported that the overall yield stress after pipe forming can be defined by the competing effect of strain hardening and BE in material. They also revealed that the competing effect was related to the microstructure and the pipe forming strain. Lodej et al. [23] predicted the mechanical history of a metal during cold pilgering of a seamless tube using 3D FEM computation based on low-cycle fatigue models because the metal undergoes a series of small incremental deformations alternatively in tension and compression.

Mutrux et al. [25] reported the cyclic softening behavior in a medium-carbon steel bar during the cross-roll straightening process. They also predicted the residual stress distribution that appears in straightened bars. Lee et al. [26] reported the BE during the multi-pass non-circular wire drawing process using low-carbon steel. They observed that the TS of the wire fabricated by the non-circular drawing process was lower than that of the conventional wire drawing process at high strain levels and suggested that this softening behavior is highly related to the BE during multi-pass non-circular wire drawing. Kumar et al. [27] found the BE during the constrained groove process press using experimental tests and numerical simulations. In addition, Narita et al. [28] observed a decrease in load during the multi-stage bolt forming process using stainless steel, and they explained the main mechanism of this phenomenon as the BE.

Therefore, this study analyzes the different behaviors of the mechanical properties of drawn and caliber-rolled wires with respect to their microstructures to determine the appropriate process between wire drawing and caliber rolling with consideration of materials and process conditions. Ferritic, pearlitic, and TWIP steels were drawn and caliber-rolled under the same process conditions, and the strength, ductility, and microstructure were compared. In addition, the different strengthening behaviors of the drawn and caliber-rolled wires were analyzed. In particular, the hardening and softening mechanisms of the caliber-rolled wire with strain were revealed using the finite element (FE) method.

## 2. Experimental Procedures and FE Analysis

### 2.1. Experimental Procedures

#### 2.1.1. Material Preparation

Two 13 mm-diameter commercial plain carbon steel rods with ferritic and pearlitic microstructures, respectively, were provided by POSCO, a steel making company in Pohang, South Korea. From a 160 mm-wide square billet, the two steels were manufactured by hot rod rolling at temperatures above 900 °C and then Stelmor cooling at an average cooling rate of 3 °C/s for the ferritic steel and 10 °C/s for the pearlitic steel. The detailed chemical compositions are listed in Table 1. Ferritic steel was used for test material in the received condition. In the pearlitic steel, the wire rod supplied after the Stelmor cooling process was austenitized at 950 °C for 0.2 h, subsequently heat treatment was conducted using a salt bath at the isothermal temperature of 560 °C to obtain a fine pearlitic structure. For TWIP steel, a 125 mm-thick ingot with the nominal composition of Fe-20Mn-0.6C-1Al was prepared through vacuum induction melting under an inert gas. The chemical composition of the ingot was confirmed by a spark optical emission spectrometer; its composition was close to its nominal composition, as shown in Table 1. Based on the thermodynamics model [29], the stacking fault energy was 23.5 mJ/m^2^, indicating that this steel was primarily deformed by slip and deformation twinning at RT [30]. To break up Mn and C segregation in the cast ingot, homogenization heat treatment was conducted at 1200 °C for 12 h, after which the ingot was hot-rolled to a 20 mm-thick plate at a finishing temperature of 950 °C. For wire drawing and caliber rolling tests, the plate was machined into round wires (13 mm diameter and 400 mm long) along the rolling direction.

#### 2.1.2. Wire Drawing and Caliber Rolling Tests

The ferritic, pearlitic, and TWIP steel wires were drawn at a drawing speed of 8.3 mm/s using a single pass-type draw bench machine, and they were cold-rolled at a rolling speed of 5 rpm using a single pass-type caliber rolling simulator at RT. To improve the surface quality of the initial wire, a chemical pickling (12.5% HCl) was carried out before wire drawing and caliber rolling tests. A spray-type lubricant (MoS_2_) was used to decrease the frictional stress during wire drawing, and no lubricant was applied in the caliber rolling process. The die and roll designs used in this experiment are listed in Table 2. An oval-round pass sequence was used during caliber rolling. That is, the wires were rolled with oval grooves at every odd pass and round grooves at every even pass. Since a horizontal-type rolling simulator was used in this test, the wire was manually rotated by an angle of 90° after each pass. The reduction in area (*R*) and nominal strain (*ε*) of the wire are calculated as follows:

(1) $$R=\frac{A_{0}-A_{1}}{A_{0}}\times 100\;\left(\%\right)$$

(2) $$\varepsilon =ln\frac{A_{0}}{A_{1}}$$

where *A*_0_ and *A*_1_ indicate the initial and final areas of the workpiece, respectively. According to the above equations, the *R* per pass was approximately 10% as listed in Table 2.

#### 2.1.3. Materials Characterizations

Uniaxial tensile tests were carried out at a strain rate of 1.0 × 10^−3^ s^−1^ using an Instron machine at RT. The tensile properties of the hot-rolled steels were measured using round-type tensile specimens (5 mm diameter and 25 mm long in gage). The drawn and caliber-rolled wires were pulled from the wire state without fabricating a tensile specimen. However, round-type tensile specimens were machined for the TWIP steel because the wire without machining tensile specimen tends to be fractured in the grip part during the test, based on the experiences by the author. All tensile tests were conducted along the rolling and drawing directions. Reduction in area (RA) was evaluated by measuring the minimum reduced diameter of the fractured tensile specimen [31] because RA is widely used as a ductility parameter for bulk forming [32,33,34].

The microstructures of the ferritic and pearlitic steels were characterized by field-emission scanning electron microscopy (FE-SEM), and that of TWIP steel was characterized by electron backscatter diffraction (EBSD). To prepare the specimens for SEM and EBSD measurements, the specimens were progressively ground with SiC papers up to 2000 grit. Subsequently, they were polished with diamond compound pastes from 6 to 1 µm, and then chemical colloidal silica suspension for approximately 1.2 ks for EBSD samples. FEG SEM with a TexSEM Laboratories (TSL) EBSD system was utilized for the EBSD data. It was operated at an acceleration voltage of 20 kV. EBSD data were obtained in an area of 120 µm × 120 µm using a step size of 0.1 µm at a sample tilt angle of 70°. Each EBSD map was post-processed using orientation imaging microscopy software. Microstructures were observed on the section of wires perpendicular to the drawing and rolling axes, i.e., C plane.

### 2.2. FE Analysis

Wires processed by caliber rolling and wire drawing have an inhomogeneous deformation with areas depending on the process parameters [4,35]. Therefore, an FE analysis was performed to evaluate the strain and stress distributions of deformed wire. The DEFORM FE software (version 11.0, Scientific Forming Technologies Corporation, Columbus, OH, USA) with a three-dimensional module was used to simulate caliber rolling and with a two-dimensional axisymmetric module for wire drawing.

The dies and rolls were considered to be rigid bodies. The workpiece was assumed to be an isotropic material with a strain hardening effect. In such a case, Hollomon’s law can simply describe the constitutive behavior of a workpiece using only the strain-hardening coefficient (*K*) and strain-hardening exponent (*n*) as follows:

*σ* = *Kε*^*n*^ (3)

Shear friction factors of 0.1 and 0.3 were selected for wire drawing [36,37] and caliber rolling [4], respectively. The initial wire with 13 mm in diameter was caliber-rolled and drawn with an R per pass of approximately 10% as shown in Table 2. Strain rate and/or temperature increase during the process were not considered in this simulation owing to the slow drawing and rolling speeds.

To reduce the calculation time and simulation cost, one-quarter of the full geometry was simulated during caliber rolling, and half of the entire geometry was modeled during wire drawing owing to their symmetric natures of the process. Approximately 15,000 hexahedral elements were used for caliber rolling and 5000 square elements were used for wire drawing.

## 3. Results

### 3.1. Mechanical Properties

**Figure 2:**
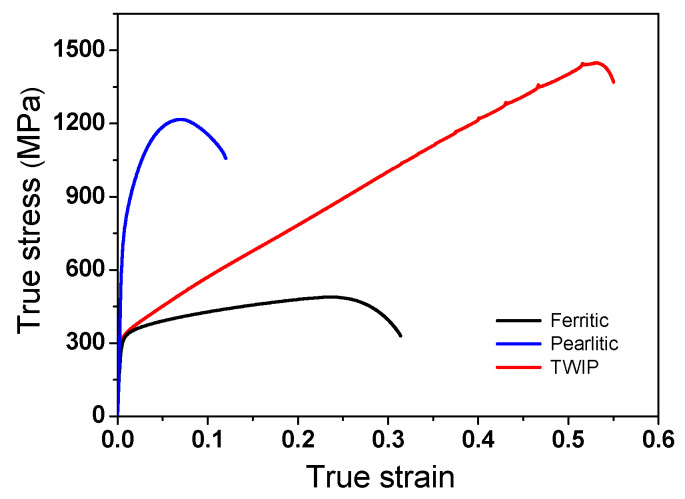
True stress-strain curves of hot-rolled ferritic, pearlitic, and TWIP steels.

The true stress-strain curves of the three hot-rolled steels are shown in Figure 2. The TWIP steel exhibited high strength and impressive ductility compared with the plain carbon ferritic and pearlitic steels primarily as a result of the deformation twins during plastic deformation [38,39,40,41,42]. Pearlitic steel exhibited the highest yield strength, and the lowest elongation.

**Figure 3:**
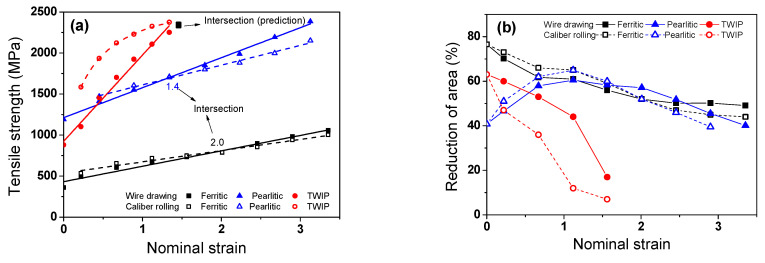
Comparison of the (**a**) TS and (**b**) RA of the three drawn and caliber-rolled steels with nominal strain.

Figure 3a shows a comparison of the TS of the drawn and caliber-rolled wires with two deformation modes as a function of nominal strain. The TS of wires fabricated by both the processes increased with nominal strain, regardless of the steel. Interestingly, in ferritic and pearlitic steels, the TS of caliber-rolled wires was higher than that of drawn wires at a low strain level, and the TS of the caliber-rolled wire was lower than that of the drawn wire at a large strain level. That is, the intersection of the TS between the drawn and rolled wires existed during the two processes. Reuter et al. [5] compared the TS of a drawn wire and caliber-rolled wire using plain carbon pearlitic steel at RT. They reported that the TS of the caliber-rolled wire was lower than that of the drawn wire, which is not consistent with the present result. In TWIP steel, the TS of the drawn wire increased linearly, whereas that of the caliber-rolled wire increased in a parabolic manner with nominal strain. That is, the strength of the caliber-rolled wire increased rapidly at a low strain level compared with that of the drawn wire. Interestingly, the caliber-rolled TWIP steel wire had considerably higher TS than that of the drawn wire. In addition, unlike in the cases of ferritic and pearlitic steels, the intersection point of TS in the drawn and rolled wires did not exist before the fracture in the TWIP steel.

Figure 3b compares the RA of the drawn and caliber-rolled wires with the steel. The RA tended to decrease with nominal strain in both processes. In TWIP steel, the RAs of the wires fabricated by caliber rolling were lower than those that underwent the wire drawing process at all strain levels. In contrast, the intersection of RA in drawn and caliber-rolled wires existed in ferritic and pearlitic steels with nominal strain, which is similar to the TS behavior. It should be noted that the intersection points of the TS between the drawn and caliber-rolled wires with strain were not related to the depletion of ductility because both the drawn and rolled wires had high RA values. In TWIP steel, the predicted intersection points of the TS between the drawn and rolled wires depended on the ductility loss because RA rapidly decreased with nominal strain. However, the parabolic increase in the TS of the caliber-rolled wire was not related to the ductility loss. 

**Figure 4:**
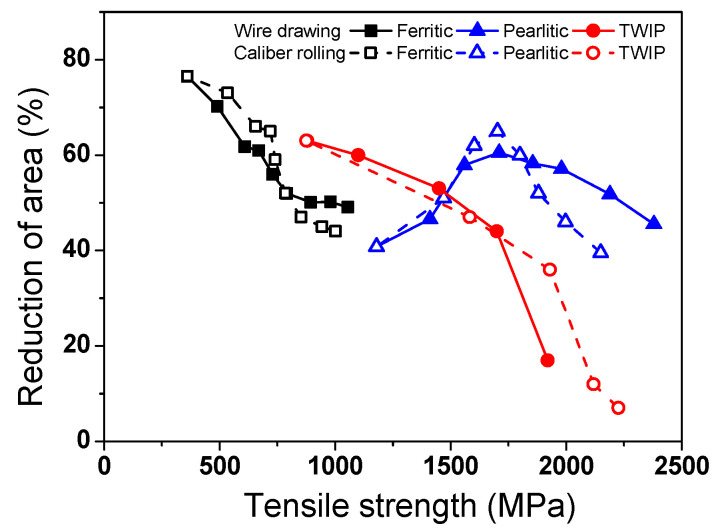
Comparison of the balance in strength and ductility of the three drawn and caliber-rolled steel wires.

Figure 4 compares the RA of the three steels as a function of TS. The RA of the ferritic and TWIP steels decreased with increasing TS. Meanwhile, the RA of pearlitic steel slightly increased up to a certain strength and then gradually decreased with strength. This unusual RA behavior of pearlitic steels has been reported by several researchers [43,44,45]. Meanwhile, the ferritic and pearlitic steels exhibited a similar behavior: caliber-rolled wire had higher RA at low strength levels and lower RA at higher strength levels compared with the drawn wire. However, the TWIP steel had an inverse relationship compared with that of the ferritic and pearlitic steels.

Overall, the caliber-rolled wire showed both hardening and softening behaviors during the process compared with the drawn wire, as shown in Figure 3a. That is, the caliber-rolled wire was highly strengthened at lower strain levels, and then softened at higher strain levels compared with the drawn wire. To utilize this phenomenon usefully in the wire, rod, and bar industries, it is necessary to reveal the hardening and softening mechanisms of the caliber-rolled wire.

### 3.2. Microstructural Evolution

**Figure 5:**
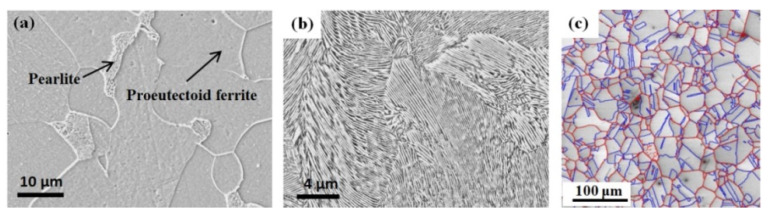
Comparison of the typical microstructures of hot-rolled (**a**) ferritic, (**b**) pearlitic, and (**c**) TWIP steels. Red and blue lines in the last figure indicate the high angle grain boundaries (−θ ≥ 15°) and Σ3 twin boundaries (−Σ3), respectively.

Micrographs of the hot-rolled three steels are compared in Figure 5. The ferritic steel was composed of a small fraction of pearlite in the proeutectoid ferrite matrix. In pearlitic steel, a lamellar structure of ferrite and cementite appeared. Annealing twins, but no deformation twins, were observed in the hot-rolled TWIP steel. These structures are general microstructures of hot-rolled or normalized ferritic, pearlitic, and TWIP steels.

**Figure 6:**
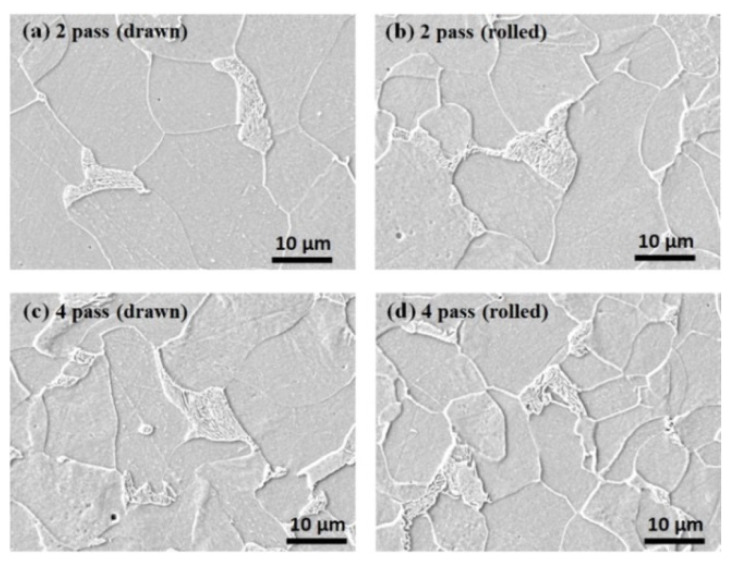
Comparison of the microstructures of the drawn and caliber-rolled ferritic steel wires with strain. Quarter region was measured.

**Figure 7:**
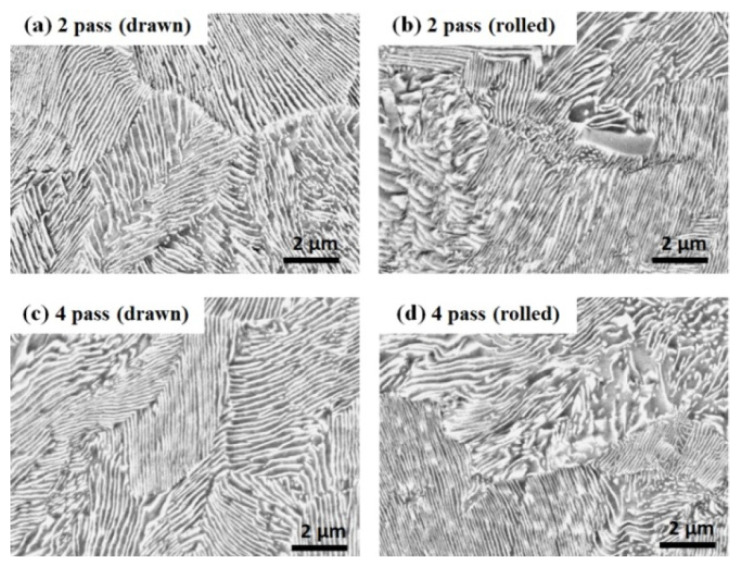
Comparison of the microstructures of the drawn and caliber-rolled pearlitic steel wires with strain. Quarter region was measured.

**Figure 8:**
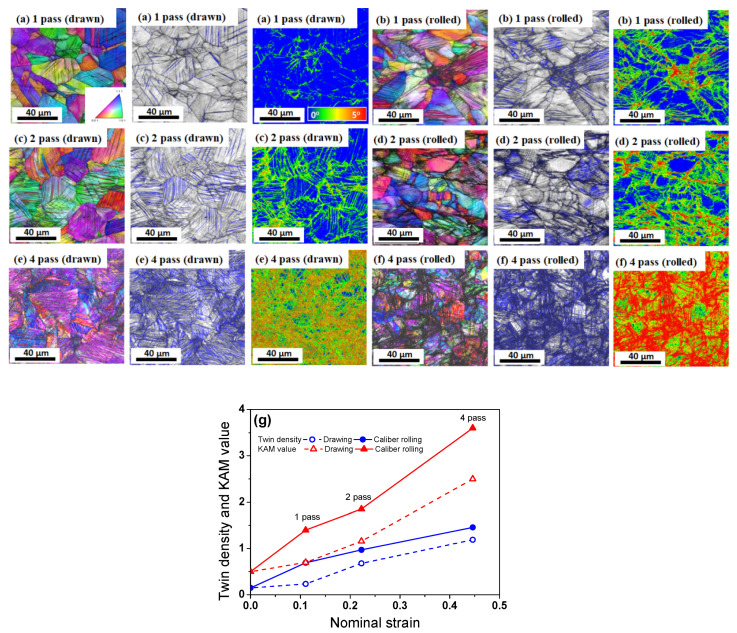
Comparison of the IPF, twin density, and KAM maps of (**a**,**c**,**e**) the drawn wire and (**b**,**d**,**f**) caliber-rolled TWIP steel wires with strain, and (**g**) variations in twin density and KAM value with the strain of the drawn and rolled wires. Quarter region was measured.

Figure 6, Figure 7 and Figure 8 show the microstructures of the three drawn and caliber-rolled steels with strain. The strength of the metals can be generally modeled using classical theories [46,47], which show that the flow stress (*σ*_*f*_) depends on the dislocation density (*ρ*) as follows:

(4) $$\sigma_{f}=\sigma_{0}+M\alpha Gb\sqrt{\rho }$$

where *σ*_0_, *M*, *α*, *G*, and *b* are the lattice friction stress, Taylor factor, a constant related to the strength of dislocation interaction, shear modulus, and Burgers vector, respectively.

The increased dislocation density primarily strengthens the ferritic steels during plastic deformation. In the case of pearlitic steel (Figure 7), the strength increases with decreasing inter-lamellar spacing [48,49]. For example, Embury and Fisher [48] proposed the following strengthening mechanism in pearlitic steel during wire drawing:

(5) $$\sigma_{f}=\sigma_{0}+\frac{k_{E-F}}{\sqrt{2}\sqrt{r_{0}}}exp\left(\frac{\epsilon }{4}\right)$$

where *k*_*E*−*F*_ and *r*_0_ are the equivalent constant of the Petch slope and the initial pearlite spacing of the wire, respectively. This equation reveals that the strength of pearlitic steel depends on the initial inter-lamellar spacing of the pearlitic structure and applied strain. The drawn and caliber-rolled wires had similar inter-lamellar spacing with strain because they had the same total *R*. However, the lamellar structure in the caliber-rolled wire was more deformed than that in the drawn wire (Figure 7), indicating that the caliber-rolled wire was highly deformed compared with the drawn wire during the process although the nominal strain was the same.

Meanwhile, TWIP steels were mainly strengthened by deformation twins formed during plastic deformation because deformation twins reduce the mean free path of mobile dislocations, resulting in the so-called dynamic Hall–Petch strengthening effect [40,50]. Although deformation twins are not the single strengthening mechanism in TWIP steels [51,52,53,54], evaluation of the twinning behaviors is necessary in TWIP steels because it is well-known that the strength increases with increasing twin volume fraction during plastic deformation [55,56,57,58,59]. Additionally, drawn and caliber-rolled wires have the same chemical composition. For example, Bouazia and Guelton [60] proposed the following model to predict the strain hardening of TWIP steel using the Kocks–Mecking model [47,61] based on Equation (4):

(6) $$\frac{d\rho }{d\varepsilon }=M\left[\frac{1}{b}\left(\frac{1}{d}+\frac{1}{2e}\cdot \frac{F}{1-F}+k\sqrt{\rho }\right)-f\cdot \rho \right]$$

where *e*, *F*, *k*, and *f* are the average twin thickness, twin volume fraction, a constant related to dislocation, and a coefficient related to the annihilation resulting from dynamic recovery, respectively. The second term on the right-hand side describes the TWIP effect or the dynamic Hall–Petch effect. The dislocation density increased with increasing *F* and decreasing *e*. Accordingly, the flow stress of TWIP steel in Equation (4) was highly related to *F* because *e* was independent of the strain [56,57,58,62]. Figure 8 compares the microstructures of the drawn and rolled TWIP steel wires with the total R. The twin volume fraction was relatively compared using the EBSD technique described in Ref. [34]. The twin density increased with the applied strain, and the caliber-rolled wire had a higher twin density than the drawn wire at the same total R (Figure 8g). According to Equations (4) and (6), the caliber-rolled wire exhibited a higher dislocation density and strength compared with the drawn wire. The kernel average misorientation (KAM) map was also compared because KAM reflects the geometrically necessary dislocation (GND) density [63,64]. In this study, the KAM value was evaluated within the third neighboring shell. The KAM maps also showed that caliber-rolled wire had a higher KAM value than the drawn wire, indicating that the GND density of caliber-rolled wire was higher than that of the drawn wire.

**Figure 9:**
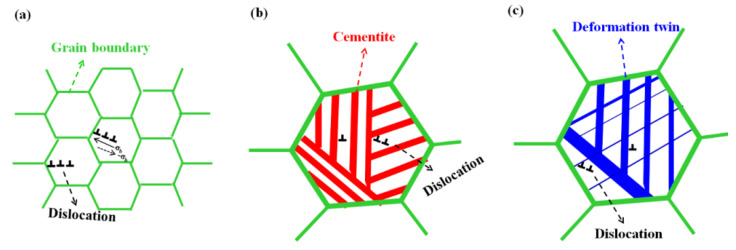
Schematic comparison of the main strengthening mechanism of (**a**) ferritic, (**b**) pearlitic, and (**c**) TWIP steels during plastic forming.

In summary, the main strengthening mechanism of the drawn and caliber-rolled wire was the restriction of the dislocation movement by grain boundaries in ferritic steel, cementite in pearlitic steel, and deformation twins in TWIP steel, as schematically depicted in Figure 9.

### 3.3. Comparison of Strain 

**Figure 10:**
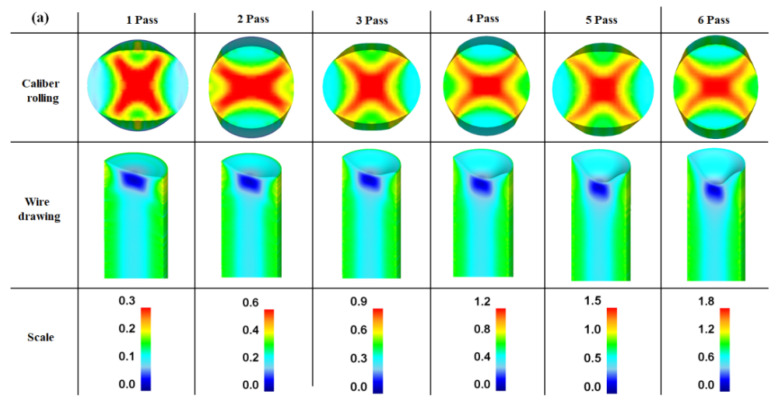

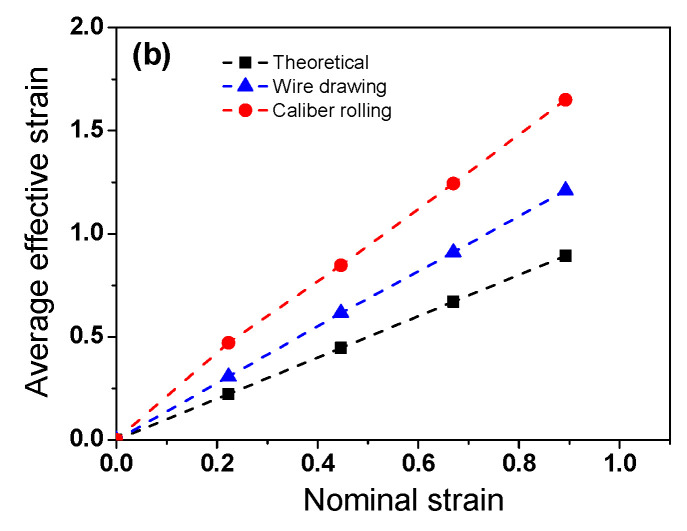
Comparison of the (**a**) contour maps of effective strain and (**b**) average effective strain of the drawn and caliber-rolled wires. Theoretical strain was obtained using Equation (2).

Figure 10a compares the von Mises equivalent strain (effective strain) contours of the caliber-rolled and drawn wires with the number of passes. For the simulation, the constitutive behavior of a workpiece was modeled based on the curve fitting of the stress-strain curve in the ferritic steel (Figure 2): *K* and *n* were determined to be 571 MPa and 0.134, respectively. In these contour maps, the vertical direction is the loading direction during caliber rolling. In the caliber-rolled wire, the center region exhibited the maximum effective strain, and the roll contact and free surface regions tended to have the minimum effective strain. Macroscopic shear bands were observed in the wires owing to the strain inhomogeneity of the caliber-rolled wire. In the drawn wires, the surface region exhibited a higher effective strain level than the central region. Figure 10b compares the average effective strain of the drawn and caliber-rolled wires as a function of nominal strain without considering any other process conditions such as imposed strain direction and rate. The theoretical strain calculated using Equation (2) was also compared. Both the drawn and caliber-rolled wires had a higher average effective strain than the nominal value. Meanwhile, the caliber-rolled wire exhibited a higher average effective strain than the drawn wire at all passes, indicating that the caliber rolling process can impose a higher strain on the specimen rather than the wire drawing process. The caliber-rolled wire had approximately 36% higher strain than the drawn wire and approximately 85% higher strain than the nominal strain.

**Figure 11:**
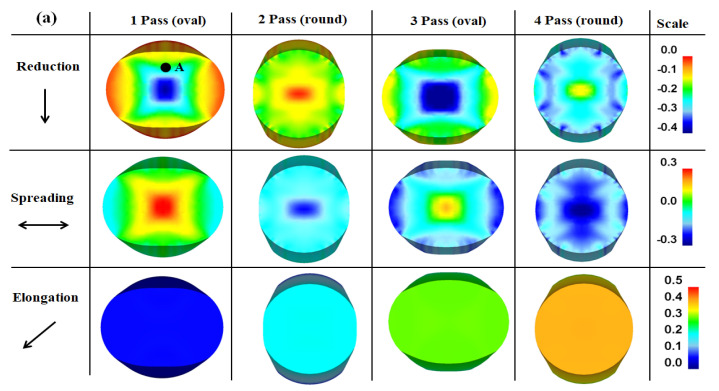

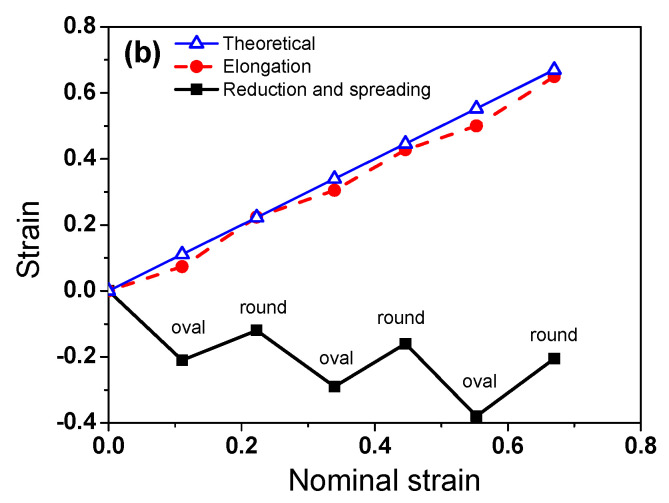
Comparison of the (**a**) strain contour with the direction of the wire and (**b**) variation in stain at point A marked in (**a**) of the caliber-rolled wire with the rolling pass. Theoretical strain was calculated using Equation (2).

**Figure 12:**
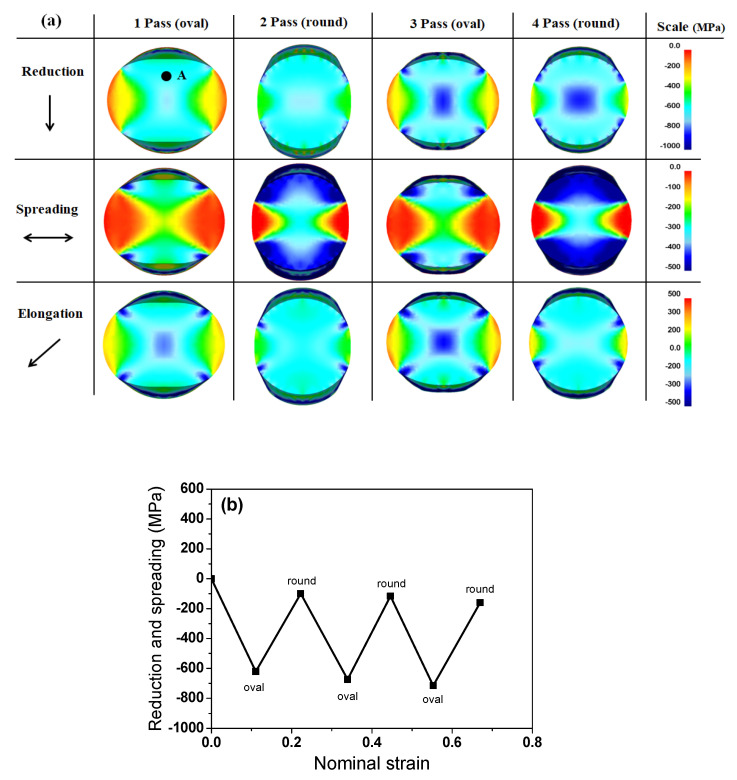
Comparison of the (**a**) stress contour with the direction of the wire and (**b**) variation in stain at point A marked in (**a**) of the caliber-rolled wire with the rolling pass.

During the conventional rolling process, the compressive stress imposed on the workpiece by the rolls deforms the workpiece in mainly three directions. In other words, the workpiece is deformed along the height, longitudinal, and width directions, and these phenomena are typically called reduction, elongation, and spreading, respectively [1]. Figure 11a shows a comparison of the strain contours against the deformation direction of the wire with the rolling pass. The reduction and spreading contours were complex with the number of rolling passes owing to the change in the loading direction per pass during the caliber rolling process. The overall reduction and spreading strains exhibited negative values with the rolling step because the cross-sectional area was reduced with the rolling steps. Accordingly, the elongation of the wire increased consistently with the rolling step, as shown in Figure 11b. The elongation of the caliber-rolled wire was similar to the nominal strain calculated using Equation (2). Based on the elongation variation in the wire with the rolling pass, it was also observed that spreading tended to occur during the oval pass and elongation primarily occurred during the round pass. Figure 11b shows the strain variation in the caliber-rolled wire along the reduction and spreading directions at point A marked in Figure 11a, that is, the quarter region of the wire. It is observed that compression and tension were applied to this region with rolling steps. For example, compressive stress or reduction was applied at the oval pass, and tensile stress or spreading was applied at the round pass. Most of the regions in the caliber-rolled wire had similar strain variations owing to the oval-round rolling sequence with a directional change in the loading in each pass, which is verified by the stress variation in the caliber-rolled wire along the wire direction as shown in Figure 12. 

**Figure 13:**
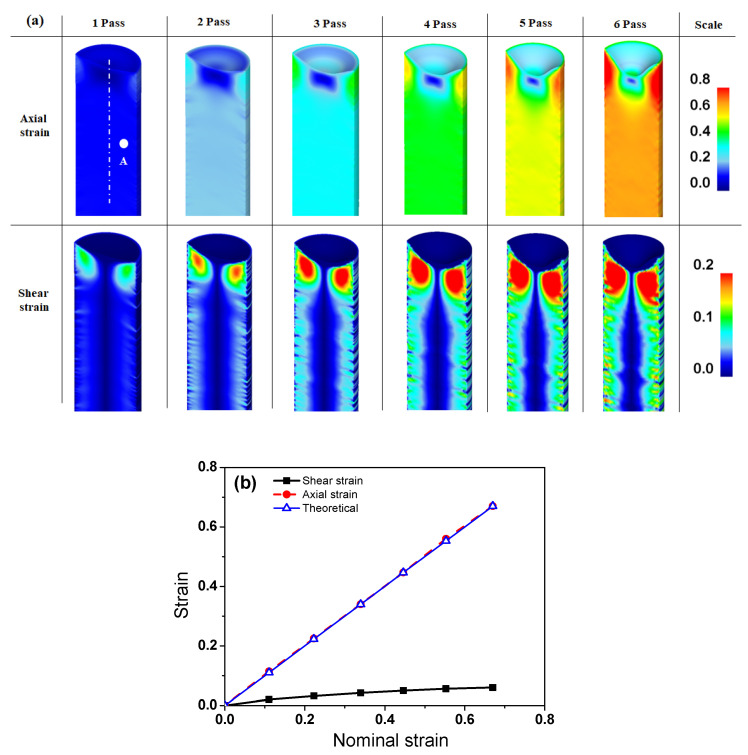
Comparison of the (**a**) axial and shear strain contours and (**b**) variation in strain at point A marked in (**a**) of the drawn wire with the drawing pass. Theoretical strain was obtained using Equation (2).

Figure 13a shows the contours of the axial and shear strains of the drawn wire with the drawing pass, and Figure 13b shows the strain variation at point A marked in Figure 13a, that is, the quarter region of the drawn wire. As expected, the axial strain was similar to the nominal strain calculated using Equation (2). The shear strain increased with the drawing step.

## 4. Discussion

Interestingly, this study showed that the caliber-rolled wire exhibited a higher strength or hardening behavior in the early deformation stage and a lower strength or softening behavior in the later deformation stage in comparison with the drawn wire regardless of the steel or microstructure. It is necessary to reveal the underlying mechanism of the hardening and softening behaviors of caliber-rolled wire. In addition, from an industrial point of view, it is helpful to suggest which process is advantageous for manufacturing wire, rod, and bar products considering the working conditions and material properties.

### 4.1. Hardening Mechanism of the Caliber-Rolled Wire

In the early deformation stage, the differences of TS in ferrite, pearlite, and TWIP steels between drawn and caliber-rolled wires were approximately 8.1%, 4.6%, and 15.7%, respectively. The higher strength of the caliber-rolled wire in the early deformation stage can be explained by the higher strain induced by caliber rolling compared with wire drawing based on the numerical simulation. As shown in the FE analysis (Figure 10b), the caliber-rolled wire had approximately 36% higher strain than the drawn wire. According to the classical plastic deformation theory [65], the total work (*w_t_*) for bulk forming includes three components: an ideal work (*w_i_*) for homogeneous deformation, frictional work (*w_f_*) at the workpiece and tool interface, and redundant work (*w_r_*). Therefore, the total work is simply evaluated as the sum of the three aforementioned works as follows:

*w_t_* = *w_i_* + *w_f_* + *w_r_* (7)

The theoretical or nominal value in Equation (2) only considered *w_i_*, resulting in the minimum value for plastic forming. The average effective strain of the caliber-rolled wire was higher than that of the drawn wire regardless of the nominal strain (Figure 10b). The caliber rolling process imposed a higher *w_r_* on the workpiece than the wire drawing process because the working conditions of caliber rolling are relatively complex. That is, the multi-pass with the change in shape and loading axis at every pass increased the internal strain or material distortion compared with the wire drawing process, leading to a higher *w_r_*, and resulting in a higher *w_t_*. The results of microstructural evolution also supported the idea that caliber rolling can impose a higher strain than wire drawing. That is, the caliber-rolled wire had a higher strength because of the highly accumulated dislocations compared with the drawn wire because the dislocation density generally increases with plastic strain. Compared with the conventional drawn wire, Muszka et al. [66], Hwang et al. [67], and Lee et al. [26] reported higher wire strengths during accumulative angular drawing, the continuous hybrid drawing process with an equal channel angular pressing die and wire drawing die, and non-circular drawing. The higher strength of the workpiece during the forming processes mentioned above compared with conventional wire drawing can be explained by the higher *w_r_* imposed on the workpiece.

### 4.2. Softening Mechanism of the Caliber-Rolled Wire

The softening behavior of the caliber-rolled wire can be related to the BE and/or low-cycle fatigue effect owing to the shape of rolls and change in the loading condition during the process. BE is the strength decrease phenomenon in reverse loading after pre-strain. For instance, a decrease in the yield strength in the compression test after the tension test was typically observed. The BE has been well-reported in ferritic [68,69], pearlitic [70,71,72], and TWIP [62,73,74] steels during tension-compression cyclic loading tests. Meanwhile, it should be noted that BE indicates a decrease in yield stress during reverse loading after forward pre-strain happens in single-dimension, whereas the softening behavior in caliber rolling was related to the low-cycle fatigue effect happens in three-dimension. However, this study did not strictly distinguish between the BE and low-cycle fatigue effect because the physical nature of the two phenomena was similar. During forward straining, the flow stress can be described as follows:

*σ_f_* = *σ_i_* + *σ_d_* + *σ_b_* (8)

where *σ_i_* means the initial yield stress of the material primarily results from the lattice friction with solid solution hardening; *σ_d_* indicates forest dislocation hardening which is mostly a result of immobile dislocations; and *σ_b_* indicates the back stress which is a primary result of mobile dislocations and is highly related to the BE. *σ_i_* and *σ_d_* are non-directional stresses because they resist deformation in both the forward and reverse stresses. In contrast, *σ_b_* resists forward deformation and aids reverse deformation, indicating that *σ_b_* from mobile dislocations moves reversibly during the change in the strain path. Therefore, the flow stress in reverse loading (*σ*_r_) can be described as follows:

*σ_r_* = *σ_i_* + *σ_d_* − *σ_b_* (9)

where *σ_b_* is primarily caused by the dislocation pileups around hard particles, such as precipitations [75], grain boundaries [76], dislocation tangles [77], twinning [73], cementite [78], and martensite [79], indicating that *σ_b_* is highly related to the GND, and hetero-structured materials have a high *σ_b_*. In pearlitic steel, dislocations gliding in the ferrite matrix are piled up at the ferrite and cementite interface (Figure 9), leading to long-range internal stress, resulting in the BE [70,78]. The deformation twins and stacking faults interact with dislocations, and these are considered the main sources of *σ_b_* or BE in TWIP steels [73,80].

During caliber rolling, the compressed quarter region or reduction region around the roll contact area in the oval pass was placed in the tensile stress state or spreading in the next round pass. In the same vein, the spreading region around the free surface of the wire in the oval pass was subjected to compressive stress in the next round pass. These sequences were repeated every rolling pass as shown in Figure 11 and Figure 12; therefore, the compressive stress and tensile stress were applied repeatedly to the wire during caliber rolling.

The BE seems to explain the softening behavior of the caliber-rolled wire; however, it cannot explain why the softening did not appear at low strain levels and appeared at high strains. Hanazaki et al. [81] reported that the BE was stronger in a material with fine grain than in a material with coarse grain. They explained that the high dislocation density within the small area of the fine grain by the plastic deformation moves back easily in the reverse deformation with the aid of *σ_b_*, leading to an increase in the BE with decreasing grain size. Sohn et al. [82] also reported that the BE was higher in fine-grained steels than in coarse-grained steels for line pipes owing to the highly activated *σ_b_* in fine-grained steel. It is clearly observed that the grain size of the ferritic and TWIP steels and inter-lamellar spacing of the pearlitic steel decreased with the total R, regardless of the process because the cross-sectional area of the wire was reduced with the number of passes, as shown in Figure 5, Figure 6, Figure 7 and Figure 8. In TWIP steel, the BE became stronger with the applied strain [74,80,83] and is typically modeled as follows:

(10) $$\sigma_{b}=M\frac{\mu \cdot b}{L}N$$

where *N* means the number of dislocations stopped at the boundaries, and *L* is the mean intercept length, which can be expressed by the following equation in TWIP steels.

(11) $$L=2e\frac{1-F}{F}$$

Accordingly, *σ_b_* increased with the twin volume fraction, indicating that *σ_b_* increased with applied strain because the twin volume fraction increased with strain (Figure 8g). In the KAM maps of TWIP steel as shown in Figure 8, a higher KAM value was observed at high angle grain boundaries and twin boundaries rather than inside the grains, indicating that the dislocations formed at these boundaries were mobile dislocations, and the *σ_b_* at points of interaction between mobile dislocations and boundaries decreases the yield stress [84]. These results demonstrated that the BE was amplified with increasing deformation because inter-lamellar spacing and twin spacing decreased with increasing deformation. That is, the number of potential obstacles to mobile dislocations increased with deformation, leading to the high effect of the *σ_b_*. Therefore, it is generally observed that the BE becomes stronger with the increasing strength of steels [22].

The above mechanism seems to explain the intersection point of the strength between the drawn and rolled wire; however, it cannot explain the different intersection points of strength with the steels. For example, the intersection strains of ferritic steel and pearlitic steel were approximately 2.0 and 1.4, respectively. In TWIP steel, although the softening behavior of the caliber-rolled TWIP steel was clearly observed at high strain levels, the intersection points did not appear before the fracture of the drawn wire. It is necessary to understand and predict when the BE appears in the strength curve during the plastic forming process to utilize the BE in the metal forming industry.

**Figure 14:**
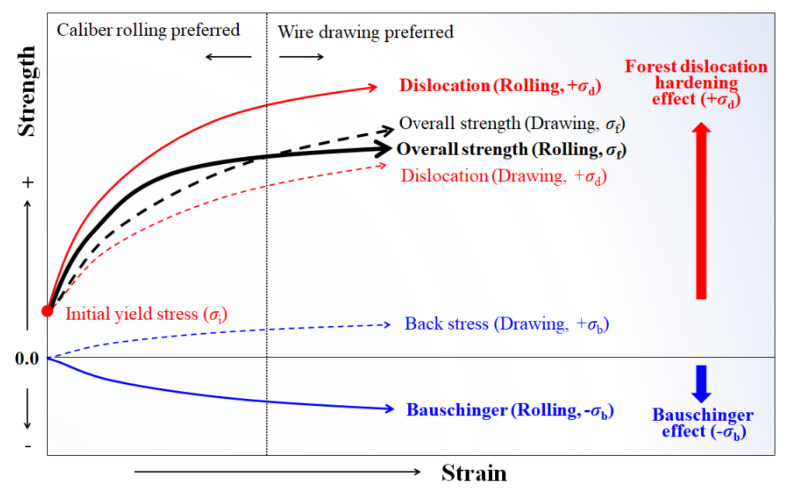
Schematic of the variation in strength of the drawn and caliber-rolled wires with strain considering forest dislocation hardening and BE.

The flow stress of the material during the plastic forming process generally depends on the hardening behavior of the material. During the caliber rolling process, the hardening behavior is governed by the competition between *σ_d_* and *σ_b_*. Figure 14 depicts the hardening and softening behavior of the material during the wire drawing and caliber rolling processes. During caliber rolling, the effect of *σ_d_* of the material can overcome the negative *σ_b_* at low strain levels owing to the higher applied strain compared with the drawn wire (Figure 10); therefore, the TS of the caliber-rolled wire was higher than that of the drawn wire. However, as the plastic deformation continued, the effect of *σ_d_* decreased, and the negative *σ_b_* effect appeared in the TS curve during caliber rolling. In such a case, the rate of strength increase seems to decrease during caliber rolling. During the wire drawing process, the BE did not appear because wire drawing is a monotonic plastic forming process, as shown in Figure 13. Therefore, the drawn wire was mainly strengthened by *σ_d_* and positive *σ_b_*. Accordingly, the intersection point of the strength between the drawn and caliber-rolled wires was observed as shown in Figure 14.

**Figure 15:**
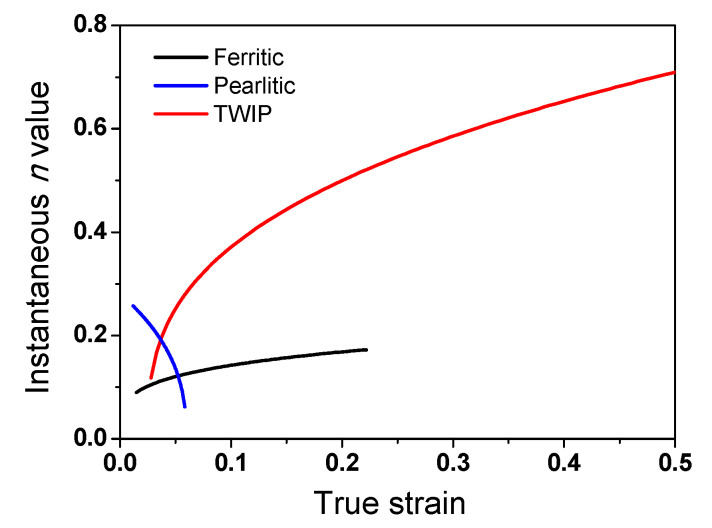
Comparison of the variation in instantaneous *n* values for the hot-rolled ferritic, pearlitic, and TWIP steels with strain.

According to the above explanation, the different intersection points of the strength between the drawn and rolled wires with the steels can be explained by comparing the flow stress curves of each steel. The flow stress is highly related to the *n* value of the material during cold forming. As the strain hardening rate or *n* value of the material is higher, the increase in strength is higher during the forming process. Therefore, the strain hardening behavior of the three steels can be evaluated by comparing the *n* values of each steel. Figure 15 shows the instantaneous *n* values of the three steels with true strain based on Figure 2. The instantaneous *n* value was obtained as follows based on Holloman’s equation in Equation (3):

(12) $$n\left(\varepsilon \right)={\frac{d\;ln\sigma }{d\;ln\varepsilon }|}_{\;\dot{\varepsilon },\;\;T=const.}$$

**Figure 16:**
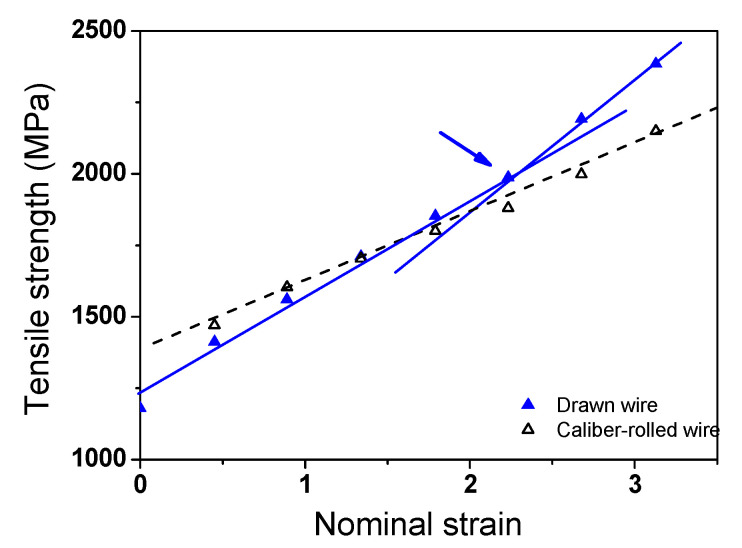
Comparison of the tensile strength of the drawn and caliber-rolled pearlitic steel with nominal strain. The arrow indicates the transition point of the increase in tensile strength of the drawn wire.

A significant difference between TWIP steel and plain carbon steels was observed: the TWIP steel exhibited a higher *n* value compared with the plain carbon steels, indicating that TWIP steel has a higher strain hardening capability compared with the ferritic and pearlitic steels. Based on Equation (9), the flow curves of the materials are affected by *σ_i_*, *σ_d_*, and *σ_b_*. Generally, *σ_d_* was relatively higher than *σ_b_* [62,80], and *σ_i_* was independent of the strain hardening rate of the materials. Accordingly, the *n* value of the tensile curve was mainly related to *σ_d_* in the metals and it was dependent on the dislocation generation rate of material. Therefore, the high dislocation generation rate of the TWIP steel delayed the appearance of *σ_b_* or the BE in the strength curve during caliber rolling compared with that during wire drawing, as shown Figure 3a. In addition, the plastic deformation mechanism of twinning in TWIP steel decreased the BE because dislocation motion with direction affected the BE in materials during deformation. In contrast, small dislocation generation rate in ferritic and pearlitic steels cannot prevent the BE from appearing in the strength curve of the caliber-rolled wires at high strain levels, as compared with the drawn wires. The critical strain for the appearance of the BE in the TS of the pearlitic steel was lower than that of the ferritic steel despite the higher dislocation generation rate as the BE in the pearlitic steel was larger than that of the ferritic steel owing to the laminar structure in pearlitic steels [85]. In addition, note that a rapid increase in the TS of the drawn pearlitic steel at a strain of 2.2 was observed, as shown in Figure 16. This strengthening behavior of the drawn pearlitic wire was also attributed to the apparent softening phenomenon in caliber-rolled pearlitic steel because caliber-rolled pearlitic steel exhibited no transition point of strength increase. Park et al. [44] reported a similar phenomenon in the drawn hyper-eutectoid steel at a nominal strain of 2.0 during wire drawing. They explained that the sudden rise in the TS of the highly drawn wire is related to the strain hardening effect of the completely aligned lamellar structure to the drawing direction during wire drawing. The present result partly supported the strengthening mechanism explained above because the lamellar structure of the wire was highly aligned during wire drawing [86] compared with caliber rolling owing to the main stress state.

Meanwhile, Lee et al. [26] observed that the TS of a ferritic steel wire fabricated by the non-circular drawing process was higher than that of the conventional wire drawing process up to the nominal strain of 1.79, and then the TS of the drawn wire with conventional dies was higher than that of the wire with non-circular dies. It was believed that the proposed hardening and softening mechanism in this study can explain the softening behavior of the drawn wire with non-circular drawing dies at high strain levels compared with the drawn wire with conventional dies.

Finally, it should be noted that the texture effect on strengthening needs to be considered because several studies have shown that the strong fiber texture generated during wire drawing increases the strength of the wire [87,88,89]. Compared with the caliber-rolled wire, the drawn wire exhibited a stronger fiber texture, and the intensity of the fiber texture increased with the drawing strain [58,90,91], indicating that the influence of the texture on strengthening increased with the drawing strain. However, this study did not consider the effect of the texture on strength. From this perspective, additional studies are necessary.

### 4.3. Selection of Caliber Rolling or Wire Drawing Process 

The caliber rolling process is the strongest candidate for replacing the wire drawing process because of its process efficiency compared with the wire drawing process. Based on the present study considering the BE, the appropriate process should be selected according to the strain hardening rate of the material and the amount of plastic deformation.

**Figure 17:**
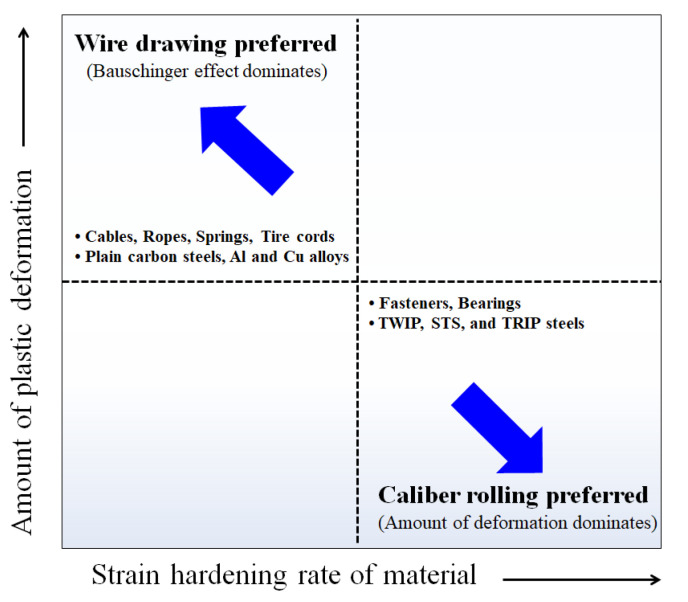
Schematic map of the appropriate process selection considering the strain hardening rate of materials and the amount of plastic deformation.

Figure 17 schematically shows the process selection map for wire drawing and caliber rolling. When the plastic deformation is small during the forming processes, such as for fasteners and bearings, caliber rolling is a better process compared with wire drawing because of both the higher strength and ductility in caliber-rolled wire (Figure 4). That is, caliber rolling produces high strength wire products with less energy compared with wire drawing, which is of benefit in wire, rod, and bar industries. In contrast, when the wires need to deform at high strain levels, such as for cables, ropes, springs, and tire cords, wire drawing is a better process than caliber rolling owing to the appearance of the BE in caliber-rolled wire. However, even for a process with a large deformation during the process, caliber rolling is an appropriate process when a material with a high strain hardening rate is used as an initial material because the BE does not appear in the strength curve of the caliber-rolled wire. For example, Hwang [2] suggested that the caliber rolling process is more suitable for making high strength metals deformed and strengthened by twinning mechanism including TWIP steels compared with the wire drawing process, which is consistent with the present result because TWIP steel has a high strain hardening rate during deformation. In summary, the material properties as well as working conditions need to be considered when choosing the optimum metal forming process in industrial fields.

## 5. Conclusions

Based on a comparative study of the caliber rolling and wire drawing processes for ferritic, pearlitic, and TWIP steels with applied strain, the conclusions are derived as follows:

1. Caliber-rolled ferritic, pearlitic, and TWIP steel wires exhibited both hardening and softening behaviors compared with the drawn wires. The hardening behavior was observed in the early deformation stage and the softening behavior appeared in the later deformation stage during caliber rolling regardless of the steel or microstructure;
2. In the early deformation stage, the differences of TS in ferrite, pearlite, and TWIP steels between drawn and caliber-rolled wires were approximately 8.1%, 4.6%, and 15.7%, respectively. The higher strengthening behavior in the early deformation stage of the caliber-rolled wire can be explained by the higher strain induced by caliber rolling compared with wire drawing, especially the higher redundant work in caliber-rolled wire. The caliber-rolled wire had approximately 36% higher strain than the drawn wire and approximately 85% higher strain than the nominal strain;
3. The softening behavior of the caliber-rolled wire in the later deformation stages is related to the BE and/or low-cycle fatigue effect originating from the roll geometry and loading conditions during caliber rolling. The different intersection points of the tensile strength between the drawn and caliber-rolled wires with the steels or microstructures can be attributed to the different strain hardening rates of each steel;
4. Between the options of caliber rolling and wire drawing processes, the appropriate process should be selected by considering the strain hardening rate of the material and the amount of plastic deformation. When the plastic deformation is small during the forming processes, such as for fasteners and bearings, caliber rolling is the better process owing to the higher strength and ductility. In contrast, when the wires need to deform at high levels, such as for cables, ropes, springs, and tire cords, wire drawing is the better process owing to the appearance of the BE in caliber-rolled wire.

## Figures and Tables

**Table 1:** Chemical compositions and heat treatment conditions of the three wire rod steels used in this experiment.

Steel Grade	Chemical Compositions in Weight Perecnt								Homogenization Conditions	
	C	Mn	Si	Cr	Al	P	S	Fe	Temp. (°C)	Time (h)
Ferrite	0.10	0.40	0.10	-	-	<0.01	<0.01	Bal.	-	-
Pearlite	0.82	0.78	0.23	0.18		<0.01	<0.01	Bal.	950	0.2
TWIP	0.60	19.94	-		1.03	<0.01	<0.01	Bal.	1200	12

**Table 2:** Process setting of dies for wire drawing and rolls for caliber rolling.

Pass Number	Wire Drawing		Caliber Rolling			Parameters	
	Wire Diameter (mm)	*R* Per Pass (%)	Roll Shape	Wire Diameter (mm)		Total *R* (%)	Nominal Strain
				Major Axis	Minor Axis		
Initial	13.00	0.00	-	13.00	13.00	0.00	0.00
1	12.30	10.48	Oval	15.60	9.70		
2	11.63	10.60	Round	11.63	11.63	19.97	0.22
3	10.97	11.03	Oval	14.40	9.30		
4	10.40	10.12	Round	10.40	10.40	36.00	0.45
5	9.86	10.12	Oval	12.80	8.20		
6	9.30	11.04	Round	9.30	9.30	48.82	0.67
7	8.80	10.46	Oval	12.20	7.30		
8	8.32	10.61	Round	8.32	8.32	59.04	0.89
*	↓	↓	↓	↓	↓	↓	↓

* The arrows in this table indicate that more tests were carried out using similar procedures.

## Data Availability

Not applicable.
